# Improved methods for genetic manipulation of the alkaliphile *Halalkalibacterium halodurans*

**DOI:** 10.3389/fmicb.2024.1465811

**Published:** 2024-09-18

**Authors:** Freya D. R. Wencker, Seth E. Lyon, Ronald R. Breaker

**Affiliations:** ^1^Howard Hughes Medical Institute, Yale University, New Haven, CT, United States; ^2^Department of Molecular Biophysics and Biochemistry, Yale University, New Haven, CT, United States; ^3^Department of Molecular, Cellular and Developmental Biology, Yale University, New Haven, CT, United States

**Keywords:** allelic replacement, anhydrotetracycline, double cross-over, HaeIII methyltransferase, homologous recombination, inducible counter-selection, *secY* antisense RNA

## Abstract

An improved approach was developed for the genetic manipulation of the Gram-positive extremophile *Halalkalibacterium halodurans* (formerly called *Bacillus halodurans*). We describe an allelic replacement method originally developed for *Staphylococcus aureus* that allows the deletion, mutation, or insertion of genes without leaving markers or other genetic scars. In addition, a protocol for rapid *in vitro* plasmid methylation and transformation is presented. The combined methods allow the routine genetic manipulation of *H. halodurans* from initial transformation to the desired strain in 8 days. These methods improve *H. halodurans* as a model organism for the study of extremophiles.

## 1 Introduction

The haloalkaliphile *Halalkalibacterium halodurans* C-125 (JCM 9153), formerly known as *Bacillus halodurans* C-125, KEGG organism code “bha” (Kanehisa et al., 2022), is a rod-shaped, Gram-positive, motile and spore-forming bacterium that was originally isolated from soil (Takami and Horikoshi, 1999; Joshi et al., 2021). *H. halodurans* is of biotechnological relevance due to its ability to produce alkali-tolerant enzymes (Takami and Horikoshi, 2000; Xu et al., 2011; Lin et al., 2013; Tekin et al., 2021) and the lantibiotic haloduracin (McClerren et al., 2006; Danesh et al., 2011). Furthermore, it serves as a model organism for alkaliphiles (Preiss et al., 2015).

Unfortunately, protocols are not available for *H. halodurans* that enable the facile genetic manipulation (e.g., deletion, insertion, or mutation) of its genomic DNA. A method previously described to manipulate the genome of *H. halodurans* employs homologous recombination (HR) and subsequent selectable marker gene excision by Xer recombination (Bloor and Cranenburgh, 2006; Wallace et al., 2012). However, this method has several disadvantages. For example, deletions can be readily made, but insertions of large regions or the introduction of point mutations or tag sequences are not easily obtained. The HR procedure also leaves a scar in the genome (*dif* site; 28 bp-long Xer recombinase recognition sequence) and the deletion of genes residing in close proximity (~30 kb) could lead to loss of the intervening region due to the presence of two closely positioned *dif* sites that lead to an undesired excision event (Bloor and Cranenburgh, 2006). This latter problem makes preparing multiple-gene knockout strains less feasible.

To overcome these disadvantages, we adapted a method of allelic replacement with inducible counter-selection previously implemented in *Staphylococcus aureus* (Bae and Schneewind, 2006; Geiger et al., 2012) for use in *H. halodurans*. In this method, the genomic region of interest is replaced by an alternative sequence provided from a plasmid. During later steps of the procedure, a counter-selection against bacteria retaining the delivery plasmid is achieved by inducing the expression of a lethal antisense transcript encoded on the plasmid. This allows for the markerless and scarless deletion of genes, the creation of point mutations, and the facile insertion of DNA sequences.

**Figure 1:**
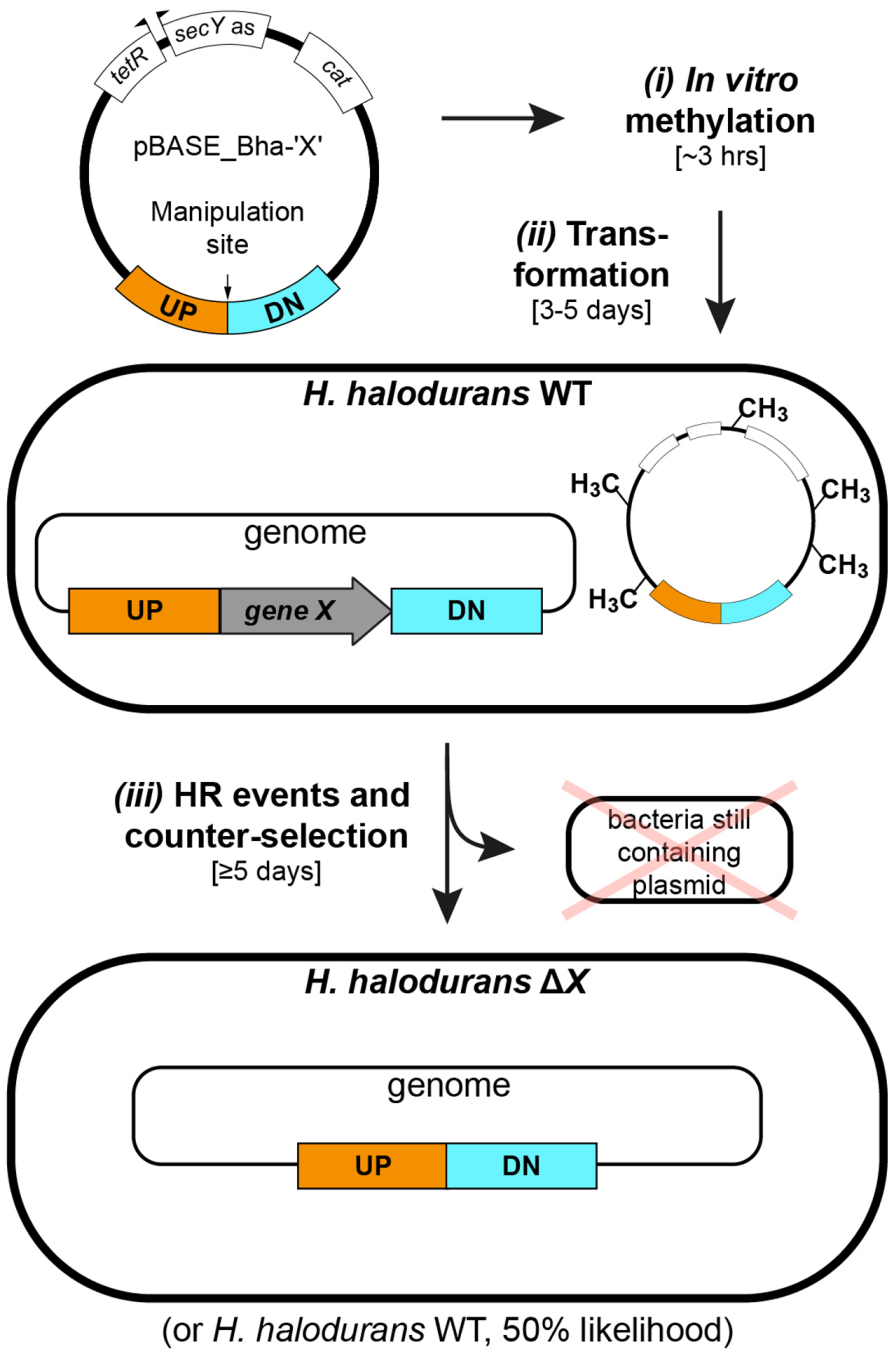
Workflow for *in vitro* plasmid methylation with subsequent transformation and allelic replacement in *H. halodurans*. Step *i*: *in vitro* methylation of plasmid DNA (approximately 3 hours). Step *ii*: *H. halodurans* protoplast transformation using *in vitro* methylated plasmid DNA (3–5 days). Step *iii*: homologous recombination (HR) events for genomic deletion (5 days). The first event involves plasmid integration into the genome. The second event involves replacement of the genomic region of interest with the plasmid-derived sequence and subsequent excision of the plasmid backbone, resulting in a genomically modified *H. halodurans* or restoration of the wild-type sequence (likelihood of 50% for both scenarios). Annotations: WT (wild type), ΔX (genomic deletion of gene X), UP (1 kb flanking region upstream of the manipulation site), DN (1 kb flanking region downstream of the manipulation site), terR (*tet* repressor), *secY* as (*H. halodurans secY* antisense sequence), *cat* (chloramphenicol acetyltransferase gene).

This adapted allelic replacement method, combined with a method for *in vitro* methylation of plasmids also described herein, enables the genomic manipulation of *H. halodurans* C-125 in 8 days from plasmid isolation to the desired strain (Figure 1). Plasmid methylation, which protects the transformed plasmid from destruction by the restriction enzymes naturally present in *H. halodurans* C-125, is achieved *in vitro* by using commercially available HaeIII methyltransferase. We demonstrate allelic replacement in *H. halodurans* by selectively creating a chromosomal deletion of the gene *yqeY* and by introducing point mutations into the *rpsU* gene to introduce two stop codons within its open reading frame. A detailed protocol for *in vitro* plasmid methylation and allelic replacement is outlined, along with possible modifications to accommodate time-restrictions and different efficiency requirements.

## 2 Materials and methods

### 2.1 Bacterial strains and growth conditions

The strains and plasmids used in this study are listed in Table 1. The pBASE_Bha vector (pFW004) is a derivative of the pBASE6 vector designed for use in *S. aureus* (Geiger et al., 2012) but adapted to *H. halodurans* by replacing the *Staphylococcus*-specific *secY* antisense sequence with one specific for targeting the *secY* mRNA of *H. halodurans* (Supplementary file S1). See section 2.6 and Table 2 for cloning strategy. pBASE_Bha is the “empty vector” not containing an insert in its multiple cloning site. All further pBASE_Bha vectors used in this study [pBASE_Bha_ΔyqeY (pFW006) and pBASE_Bha_rpsU** (pFW032)] are derivatives of pBASE_Bha. *Escherichia coli* NEB5α (#C2987, New England Biolabs, Ipswich, MA, United States) transformed with pBASE_Bha-based vectors were grown in Luria Bertani (LB) Broth (J75852-A1, Thermo Fisher Scientific, Fair Lawn, NJ, United States) medium supplemented with carbenicillin (Carb, #C1389, Sigma-Aldrich, St. Louis, MO, United States) at a final concentration of 100 μg mL^−1^ for plasmid isolation. *E. coli* TOP10 + pPAMC125 cells transformed with pBASE_Bha-based vectors for *in vivo* methylation were grown in LB medium supplemented with chloramphenicol (Cm, #C0378, Sigma-Aldrich) at a final concentration of 5 μg mL^−1^ in addition to Carb for plasmid isolation. A 3 mg mL^−1^ working stock of Cm was established in ≥99.8% ethanol from a 100 mg mL^−1^ stock (also in ≥99.8% ethanol). If not stated otherwise, *H. halodurans* was grown in LB Broth medium supplemented with Na_2_CO_3_ to a final concentration of 94.4 mM to obtain a pH of 10. The LB broth medium was set up with ddH_2_O to 90% of the final volume, autoclaved and then supplemented with sterile-filtered Na_2_CO_3_ solution to the final volume. If not stated otherwise, *E. coli* and *H. halodurans* were cultivated at 37°C on solid media and with shaking at 220 rpm in liquid media.

**Table 1:** Bacterial strains and plasmids.

Strains	Description	References
*E. coli* NEB5α	Commercially available *E. coli* DH5α derivative, T1 phage resistant, endA deficient, common cloning strain for plasmid isolation	NEB #C2987I
*E. coli* TOP10 + pPAMC125	*E. coli* TOP10 (commercially available) with pPAMC125 vector for *in vivo* methylation of plasmids to be transformed into *H. halodurans* C-125	Wallace and Breaker (2011)
*H. halodurans* C-125	Rod-shaped, Gram-positive, motile and spore-forming, alkaliphile, halophile, and originally isolated from soil	Takami and Horikoshi (1999), Joshi et al. (2021)
**Plasmids**		
pBASE6	ori pE194ts (temperature-sensitive); ori ColE1; G+/G- shuttle; *cat*, *bla*; *S. aureus* antisense *secY* under P_xyl/tet_ promoter	Geiger et al. (2012)
pBASE_Bha (pFW004)	ori pE194ts (temperature-sensitive); ori ColE1; G+/G- shuttle; *cat*, *bla*; Bha antisense *secY* under P_xyl/tet_ promoter	This study	pBASE_Bha_yqeY+ 1kb _fl (pFW005)	pFW004 with *yqeY* and 1 kb flanking regions of *yqeY*	This study
pBASE_Bha_ΔyqeY (pFW006)	pFW004 with 1 kb flanking regions of *yqeY*	This study
pBASE_Bha_rpsU** (pFW032)	pFW004 with rpsU** and 1 kb flanking regions (rpsU**: insertion of two early stop codons, Glu3* and Arg5*)	This study

**Table 2:** Oligonucleotides.

Purpose	Template	Name	Sequence
**Verification of successful cloning and transformation**			
		pBASE_MCS_F	GATGCCTCAAGCTAGAGAGTCATTACC
		pBASE_MCS_R	CCATGTATTCACTACTTCTTTCAAACTCTCTC
**Cloning pBASE_Bha (pFW004)**			
3.7 kb fragment (fr.) pFW004	pBASE6	FW039	GGCGAGTTACATGATCCCCCATGTTGT
		FW041	GCTAAGGGAGTCGGAAACCCATCAAGCTTATTTTAATTATACTCTA
2.4 kb fr. pFW004	pBASE6	FW040	GGGGGATCATGTAACTCGCCTTGAT
		FW042	CATTAGATCACCTCAGTTCCTTAAGGGTAACTAGCCTCGCCG
*secY* antisense fr. (512 bp) pFW004	Genomic DNA *Bha*	FW045	GCTAGTTACCCTTAAGGAACTGAGGTGATCTAATGTTCCGAACGAT
		FW046	ATAAGCTTGATGGGTTTCCGACTCCCTTAGCTGTAATCTGCT
**Cloning pBASE_Bha_yqeY + 1kb_fl (pFW005)**			
0.9 kb fr. pFW005	pFW004	FW053	CGGCGATTGCCTTCCGCTGCACTGCGATGAGT
		FW039	See above
5.7 kb fr. pFW005	pFW004	FW054	GCTAATCATTCCTTGCATGCCTGCAGAACGGATTGTTG
		FW040	See above
*yqeY* + 1 kb flanking insert (2.3 kb)	Genomic DNA *Bha*	FW051	CAGTGCAGCGGAAGGCAATCGCCGTTGTGGCT
		FW052	GCAGGCATGCAAGGAATGATTAGCGGATTTGTCACAA
**Cloning pBASE_Bha_ΔyqeY (pFW006)**			
6.6 kb fr. pFW006	pFW005	FW059	GTGTTGAAATTGAACAATCCGACGTCTTGTCATTGGACAGG
		FW040	See above
1.9 kb fr. pFW006	pFW005	FW056	GGATTGTTCAATTTCAACACCCTCTTTTATTTAGAACTTACGCTT
		FW039	See above
Cloning pBASE_Bha_rpsU** (pFW032)			
7.2 kb fr. pFW032	pFW005	FW159	GGGAAAGAAAATGGCATAAACTTGAGTTCGTAAAAACGA
		FW040	See above
1.7 kb fr. pFW032	pFW005	FW160	CGATTCGTTTTTACGAACTCAAGTTTATGCCATTTTCT
		FW039	See above
Amplification of genomic region of *yqeY/rpsU* (for screening of Δ*yqeY* and rpsU**)			
		FW057	CCCCTAACATAATGGCAAT
		FW058	GGAATAGAGCTCAAGCACAA

### 2.2 Growth conditions for allelic replacement procedure

For the allelic replacement process*, H. halodurans* was grown on LB agar (#113002232, MP Biomedicals, Santa Ana, CA, United States) or in LB media at different pH values noted in section 3.2. Either Na_2_CO_3_ was added to a final concentration of 94.4 mM (for pH 10), 100 mM NaHCO_3_ (for pH 8.5 LB liquid media) or 50 mM NaHCO_3_ and 50 mM NaCl (for pH 8.5 LB agar, which avoids NaHCO_3_ crystallization). Sterile-filtered solutions of Na_2_CO_3_ (944 mM) or NaHCO_3_ (1 M) were added to the autoclaved, volume-adjusted media and agar. Media and agar were supplemented with Cm (final concentration of 3 μg mL^−1^) or anhydrotetracycline (ATc, #10009542, Cayman Chemical Company, Ann Arbor, MI, United States) (final concentration of 100 ng mL^−1^) as detailed elsewhere. A 100 μg mL^−1^ ATc working stock in 70% ethanol was prepared from a 2 mg mL^−1^ stock (also in 70% ethanol) and stored at −20°C with protection from light.

### 2.3 Plasmid isolation

Plasmid DNA for *in vitro* methylation or ensuing transformation into *E. coli* + pPAMC125 was isolated from *E. coli* NEB5α according to the manufacturer’s instructions using the QIAprep® Spin Miniprep Kit (#27106, Qiagen, Hilden, Germany). For *in vivo* methylation of the plasmid DNA, the construct was transformed into chemically competent bacteria of the engineered *E. coli* + pPAMC125 strain (Wallace and Breaker, 2011) using standard heat shock procedures. Plasmid DNA was subsequently isolated as described above. To determine the approximate concentration of *in vivo* methylated plasmid in the preparation, the DNA concentration determined by A_260_ spectrophotometric measurement was divided by two, assuming that the ratio of the pPAMC125 and the plasmid of interest is 1:1 in the plasmid preparation.

### 2.4 *In vitro* plasmid methylation

**Figure 2:**
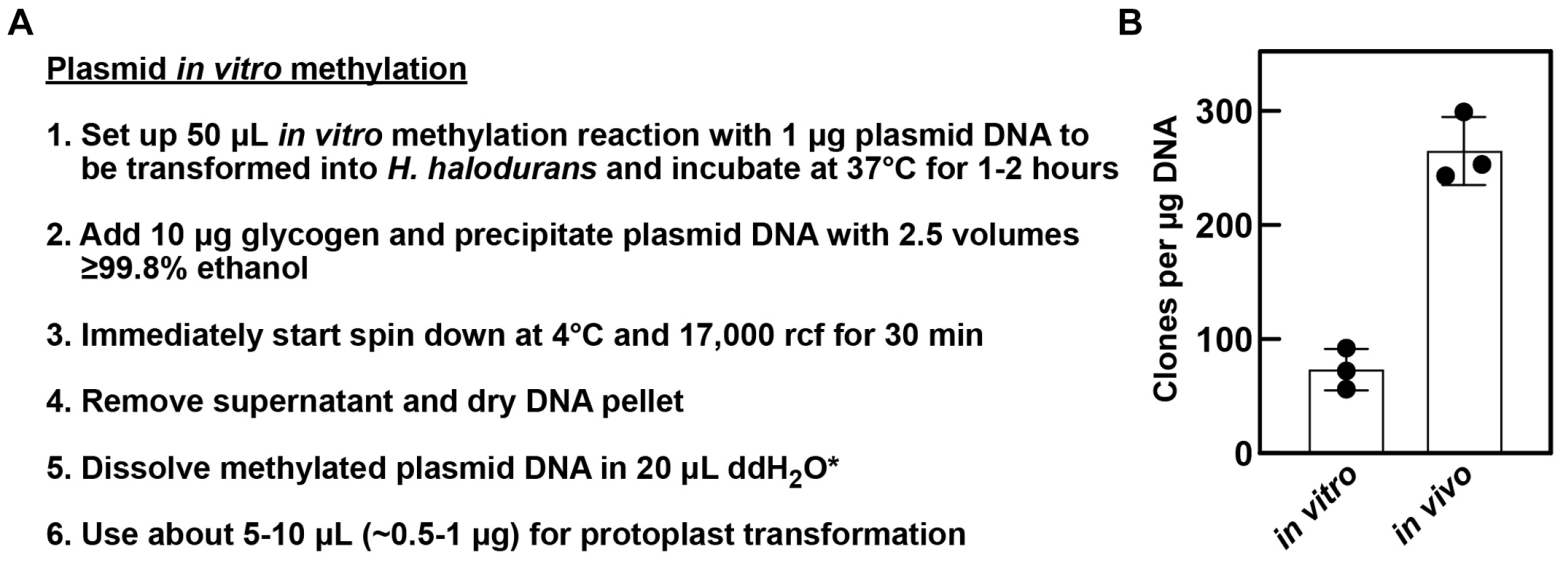
*In vitro* methylation of plasmids. **(A)** Workflow of plasmid preparation after *in vitro* methylation for ensuing protoplast transformation of *H. halodurans*. *In step 5, methylated plasmid DNA can be dissolved in 20 μL 1× SMM solution instead of water (see section 2.5 for Bha protoplast transformation method details). **(B)** Comparison of transformation efficiency of *in vitro* and *in vivo* methylated plasmid DNA. *H. halodurans* wild-type protoplasts were transformed with ~500 ng (*in vitro*) and ~ 375 ng (*in vivo*) pBASE_Bha plasmid DNA, respectively, either methylated *in vitro* or isolated from the engineered *E. coli* + pPAMC125 strain that performs the methylation *in vivo*. Obtained transformants values were normalized as clones per μg DNA. Bars represent the average clones per μg DNA resulting from three independent transformations, each. Error bars depict standard deviation.

HaeIII methyltransferase of *Haemophilus aegyptius* (#M0224S, New England Biolabs) was used for *in vitro* methylation of plasmid DNA as described in Figure 2A and in the text. Glycogen, RNA grade (#R0551, Thermo Fisher Scientific) was used to increase plasmid DNA precipitation.

### 2.5 Transformation of *Halalkalibacterium halodurans*

Protoplast transformations of *H. halodurans* to introduce plasmids were conducted as described in Wallace and Breaker (2011). Briefly, exponentially growing *H. halodurans* cells cultured in LB (pH 10) were harvested from 6 mL at 3,000 rcf for 10 min, the cell pellet was resuspended in 0.1 volumes of ASMMP buffer [1 volume of 4× BD Difco^™^ Dehydrated Culture Media: Antibiotic Medium 3, pH 7.5 (#DF0243-17-8, Thermo Fisher Scientific) and 1 volume of 2× SMM solution [1 M sucrose, 0.04 M maleate, 0.04 M MgCl_2_ (pH 6.5)]] as described elsewhere (Chang and Cohen, 1979; Kudo et al., 1990). Then cells were treated with 5 mg mL^−1^ lysozyme from chicken egg white (#4403, MilliporeSigma, Burlington, MA, United States) for 1 h at 37°C with agitation at 100 rpm, pelleted at 2,600 rcf for 10 min, once washed with ASMMP buffer and resuspended in ASMMP buffer to an optical density (OD) at 600 nm of 10. 500–1,000 ng methylated plasmid DNA was mixed with an equal volume of 2× SMM solution (see above), then 150 μL of the protoplast preparation was added to the plasmid DNA mix, incubated for 1 min at room temperature. Following, the protoplast plasmid DNA mix was transferred into 450 μL of a 30% (w/v) polyethylene glycol 8000 (PEG) (#PR-V3011, Thermo Fisher Scientific) solution (PEG dissolved in 1× SMM buffer), gently mixed by inversion and incubated for 2 min at room temperature. Then, 1.3 mL ASMMP buffer was added and gently mixed by inversion. Protoplasts were pelleted at 2,600 rcf for 10 min, very gently resuspended in 300 μL ASMMP buffer and incubated for 90 min at 30°C with agitation at 100 rpm. After recovery, protoplasts were pelleted in a table-top centrifuge for 1 min, 150 μL of the supernatant was discarded and cells were gently resuspended in the remaining volume. Subsequently, the suspension was carefully plated on M-NB regeneration medium agar (1 volume 2× Nutrient Agar, 1 volume 1 M sodium succinate, pH 7.8). 50 mL of 2× Nutrient Agar contained 0.5 g peptone (#211910, Polypeptone^™^ Peptone), 0.3 g beef extract (#211520, Bacto™ Beef Extract, desiccated) and 1.5 g agar (#DF0140-01-0, BD Bacto^™^ Dehydrated Agar) (all purchased from Thermo Fisher Scientific). After autoclaving of the 2× Nutrient Agar an equal volume of the 1 M sodium succinate solution was added and agar mix was supplemented with Cm (final concentration of 3 μg mL^−1^). Agar plates were incubated at 30°C for ≥2 days until colonies appeared. Colony PCRs to check for successful transformation using primers pBASE_MCS_F and pBASE_MCS_R primers (see Table 2 for all oligonucleotides) were conducted using the DreamTaq Green PCR Master Mix (2×) (#K1082, Thermo Fisher Scientific) as recommended by the manufacturer. Colony PCRs were of poor quality or failed with other commercially available PCR kits.

### 2.6 Cloning of pBASE_Bha vectors

For generation of plasmids pFW004-006 and pFW032 (Table 1) “*in vivo E. coli* cloning” (iVEC)’ was conducted as described previously (Wencker et al., 2021) using a previously reported strategy (Nozaki and Niki, 2019). In brief, DNA fragments overlapping by ~25–35 bp were generated by PCR, DpnI-treated (#FD1703, Thermo Fisher Scientific), purified using a QIAquick^®^ PCR Purification Kit (#28106, Qiagen) and transformed into chemically competent *E. coli* NEB5α cells in the presence of 1× Quick Ligation Reaction Buffer (part of M2200S, B2200SVIAL, New England Biolabs) following a standard heat-shock protocol. For example, PCRs to generate pBASE_Bha (pFW004) were conducted by (i) amplifying the pBASE6 vector as two fragments overlapping in the AmpR cassette and with overlaps to the Bha antisense *secY* sequence and (ii) amplifying the Bha antisense *secY* sequence from genomic DNA of *H. halodurans*.

### 2.7 Verification of successful allelic replacement

Colony PCRs to check for successful chromosomal modification (either deletion of *yqeY* or introduction of point mutations into *rpsU*) using appropriate primers (Table 2) were performed according to standard procedures. Primers should be targeted to bind approximately 20 to 40 bp up- and downstream of the ~1 kb flanking regions to ensure amplification arises only from genomic sequences and not from the plasmid.

## 3 Results

### 3.1 *In vitro* plasmid methylation with a HaeIII methyltransferase can replace time-consuming *in vivo* methylation

To reduce both the time and effort needed to genetically manipulate *H. halodurans* C-125, we revised the protoplast transformation method previously established in our laboratory (Wallace and Breaker, 2011). A key step in this protocol is the *in vivo* methylation of the plasmid of interest in an engineered *E. coli* strain prior to transformation. This step overcomes the ability of *H. halodurans* to degrade a foreign plasmid via DNA restriction enzymes naturally encoded in its genome. The engineered *E. coli* strain harbors the pPAMC125 vector with genes for two DNA adenine-specific methyltransferases (BH4003 and BH4004) and a gene for HaeIII DNA 5-cytosine methyltransferase (BH3508). These enzymes methylate plasmids passaged through the strain, which enables protection from *H. halodurans* C-125 restriction endonucleases upon transformation. This *in vivo* methylation step improves transformation efficiency into *H. halodurans* C-125 by up to 1,000-fold relative to untreated plasmids isolated from standard *E. coli* cloning strains (Wallace and Breaker, 2011). However, *in vivo* methylation requires additional time and work to transform the plasmid into the *E. coli* + pPAMC125 strain and subsequent plasmid isolation, which takes ~3 days.

An important DNA methyltransferase for *in vivo* methylation is a type II DNA 5-cytosine methyltransferase that is encoded by the prophage gene BH3508 (M.BhaII, AYT26_RS17595) (Roberts et al., 2003). BH3508 and its corresponding restriction endonuclease BH3509 are embedded in a prophage within the *H. halodurans* C-125 genome and are absent in other *H. halodurans* strains such as the NCBI reference isolate LB-1 or the strain DSM 497 (protein BLAST analysis, data not shown). These methyltransferases modify the internal cytosine residue at the C5 position of the sequence GGCC. The homolog HaeIII methyltransferase of *Haemophilus aegyptius* is commercially available (Slatko et al., 1988). Therefore, we reasoned that purified HaeIII methyltransferase could be exploited to prepare methylated plasmid DNA *in vitro* for subsequent transformation into *H. halodurans* with high efficiency. Previous studies in, e.g., *Helicobacter pylori* and *Streptomyces griseus* successfully employed *in vitro* methylation of plasmids to increase transformation efficiency (Donahue et al., 2000; Kwak et al., 2002). To demonstrate this method for *H. halodurans* with the HaeIII methyltransferase, *in vitro* methylation reactions were established with 1–2 μg plasmid DNA following the manufacturer’s instructions for methylation of genomic DNA (Figure 2A). For comparison of transformation efficiencies, we transformed the *in vivo* and *in vitro* methylated pBASE_Bha vector in parallel into *H. halodurans* using the protoplast transformation method (Wallace and Breaker, 2011). Although, transformation with the *in vitro* methylated plasmid is about four times less efficient than with the *in vivo* methylated vector (Figure 2B), it commonly yielded nearly 100 transformants per μg of plasmid DNA, and no clones were observed to be false positives (data not shown). Thus, *in vitro* methylation of plasmids allows for substantial transformation success with *H. halodurans* in less than 1 day compared to 3 days for the *in vivo* methylation process.

### 3.2 Allelic replacement with inducible counter-selection adapted to *Halalkalibacterium halodurans*

Next, we modified the allelic replacement procedure previously used with *S. aureus* to generate genomic alterations in *H. halodurans*. The DNA construct used is an *E. coli*/Gram-positive shuttle vector with a temperature-sensitive origin of replication (pE194ts), a chloramphenicol resistance cassette (*cat* gene), and a “payload” sequence designated to replace the chromosomal sequence. This payload region carries ~1 kb of sequences on each flank of the deletion, insertion, or mutation site. This extensive homology between the plasmid and the target site for genomic modification promotes HR at the desired location. The initial recombination event is expected to integrate the plasmid into the genome at the desired location. A second recombination event then either leads to the (allelic) replacement of the original targeted DNA region with the plasmid-derived sequence or restores the original genomic wild-type sequence and removes the plasmid sequence.

The vector also contains an antisense sequence of the essential gene *secY*. Transcription of this “*secY* as” RNA is regulated by an anhydrotetracycline (ATc)-inducible promotor, and its production enables counter-selection against bacteria still containing the plasmid. Counter-selection is necessary to isolate transformants that have eliminated the plasmid vector. Isolation of plasmid-free cells by exposure to ATc occurs because the antisense RNA forms a duplex with the *secY* mRNA and prevents its translation. SecY is a membrane protein and a component of the SecYEG translocase necessary for protein secretion (Oswald et al., 2021). Protein secretion, and therefore *secY* expression, is essential for bacterial growth and survival (Ji et al., 2001).

**Figure 3:**
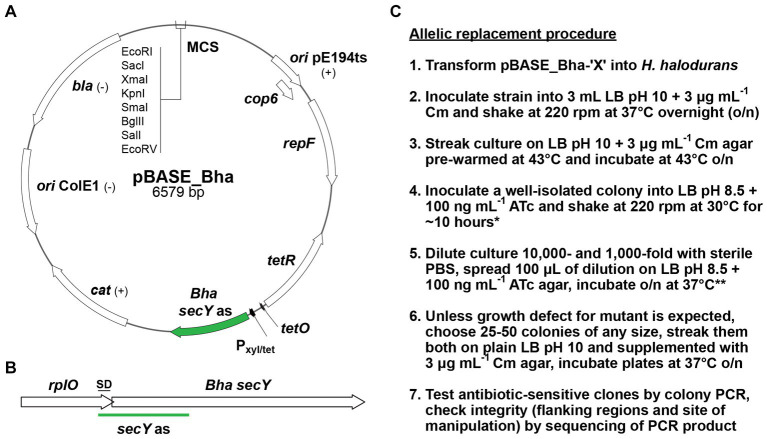
pBASE_Bha vector map, *H. halodurans secY* locus, and allelic replacement workflow. **(A)** Map of pBASE_Bha depicting the Gram-positive temperature sensitive origin of replication (*ori* pE194ts) with the *repF* and *cop6* genes. Annotations: (+) or (–) indicates functions in Gram-positive or Gram-negative bacteria. *tetR* (*tet* repressor), P_xyl/tet_ (hybrid *xyl* promoter with two *tet* operator sites), *Bha secY* as (*H. halodurans secY* antisense sequence), *cat* (chloramphenicol acetyltransferase gene), *ori* ColE1 (Gram-negative origin of replication), *bla* (β-lactamase gene), MCS (multiple cloning site with selected restriction enzymes). **(B)** The site of *secY* antisense (as) interaction with the *secY* locus of *H. halodurans* (*Bha*). The 3′ region of the upstream gene *rplO* and the *secY* Shine-Dalgarno (SD) sequence overlap. **(C)** Workflow for the allelic replacement procedure. Annotations: Cm (final concentration of 3 μg mL^−1^ chloramphenicol), ATc (final concentration of 100 ng mL^−1^ anhydrotetracycline). *If necessary, cultures at step 4 can be incubated overnight. **When cultures at step 4 were incubated overnight, plates at step 5 should be incubated only at 30°C instead of 37°C to avoid overgrowth.

If the desired genetic changes occur, the plasmid will have integrated into the genome at the locus of interest via homologous recombination at the non-permissive temperature for plasmid replication, i.e., 43°C. Subsequently, cultivation of the bacteria in the presence of ATc allows for the selection of bacteria that have undergone a second homologous recombination event to eliminate the non-payload portion of the vector from the chromosome (Figure 1). Unless the portion of the plasmid carrying the *secY* antisense RNA gene is eliminated or mutated, ATc treatment will be lethal to the cell. Importantly, to adapt the *S. aureus* pBASE6 vector for use in *H. halodurans*, we replaced the *Staphylococcus*-specific *secY* antisense sequence with one specific for targeting the *secY* mRNA of *H. halodurans* to create pBASE_Bha (Figure 3A; Supplementary file S1). The sequence spans the first half of *secY* (BH0154, AYT26_RS00900) and includes its Shine-Dalgarno (SD) region (Figure 3B).

In addition to preparing the plasmid for transformation into *H. halodurans*, the allelic replacement procedure was optimized for the high pH media preference of this species. With pH 10 growth media, 50 ng mL^−1^ ATc did not sufficiently induce *secY* antisense transcription to permit counter-selection. Our initial attempt to delete a gene (*yqeY* [BH1355, AYT26_RS07050]) from the genome of *H. halodurans* using the allelic replacement method with inducible counter-selection was inefficient. Of 95 colonies that emerged after counter-selection (step 5, Figure 3C) on LB pH 10 agar supplemented with 50 ng mL^−1^ ATc, only two were chloramphenicol-sensitive (~2%) indicating that the remaining 93 still contained the pBASE_Bha-based vector and had not undergone the second homologous recombination event. Only one of the two chloramphenicol-sensitive candidates contained the desired deletion (Table 3). From previous experience with the counter-selection method in *S. aureus*, this high number of failed counter-selections was unexpected, and a higher number of initial candidates was expected. We speculated that uptake of active ATc was insufficient at pH 10, perhaps because it is insufficiently stable (recommended pH for storage is 7.2). To compensate, ATc concentration was increased to 100 ng mL^−1^ and pH 8.5 growth medium was used. These conditions permitted robust bacterial growth while yielding sufficient *secY* antisense transcript production to enable counter-selection. We attempted to obtain single chromosomal deletions for 10 additional genes with the adjusted pH and ATc concentration. The efficiency of counter-selection now ranged from ~6 to 57%, with the majority of attempts (8 out of 10) yielding in at least 20% of chloramphenicol-sensitive clones (Table 3).

**Table 3:** Rates of successful counter-selections and gene deletions.

Gene	Cm-sensitive clones (total streaked)	Cm-sensitive clones [~ %]	Clones with desired deletion	Cm-sensitive clones with deletion [%]
*atpF* (ATP synthase, F0 complex, subunit B)	4 (13)	31	1	25
BH0616 (unknown function)	3 (13)	23	3	100
*bmrCD* (multidrug ABC transporter)	4 (13)	31	1	25
*cydA* [cytochrome bd ubiquinol oxidase (subunit I)]	20 (35)	57	5	25
*efp* (elongation factor P)	3 (13)	23	2	~66
*flhF* (signal recognition particle-like GTPase)	4 (13)	31	1	25
*rsbT* (PP2C activator, protein serine kinase)	25 (87)	29	1	4
*rocA2* (1-pyrroline-5-carboxylate dehydrogenase)	1 (18)	6	1	100
*yhcR* (extracellular non-specific endonuclease, RNase)	1 (13)	8	1	100
*yhcX* (putative C-N hydrolase)	5 (13)	54	1	20
*yqeY* (unknown function)	2 (95)*	2*	1*	50*

Cm: chloramphenicol, *counter-selection performed in LB with pH 10 and 50 ng mL^−1^ ATc (initial attempt).

The complete workflow for the allelic replacement procedure is briefly outlined below (Figure 3C), with procedural recommendations and possible modifications highlighted. All steps involving ATc should be performed with minimal light exposure to prevent degradation of photosensitive ATc (Baumschlager et al., 2020). LB (pH 8.5) + ATc (100 ng mL^−1^) plates should be prepared fresh by spreading the required volume of ATc (delivered in sterile phosphate-buffered saline [PBS]) on the agar. When step 4 is performed with a short incubation (~10 h), plates for step 5 should be incubated at 37°C instead of 30°C to ensure sufficient colony size the next day. For screening on agar supplemented with chloramphenicol in step 6, it is recommended that the incubation proceeds at least overnight at 37°C to exclude false negative results (no growth on Cm despite presence of vector) due to potentially slightly delayed growth in the presence of the antibiotic.

**Figure 4:**
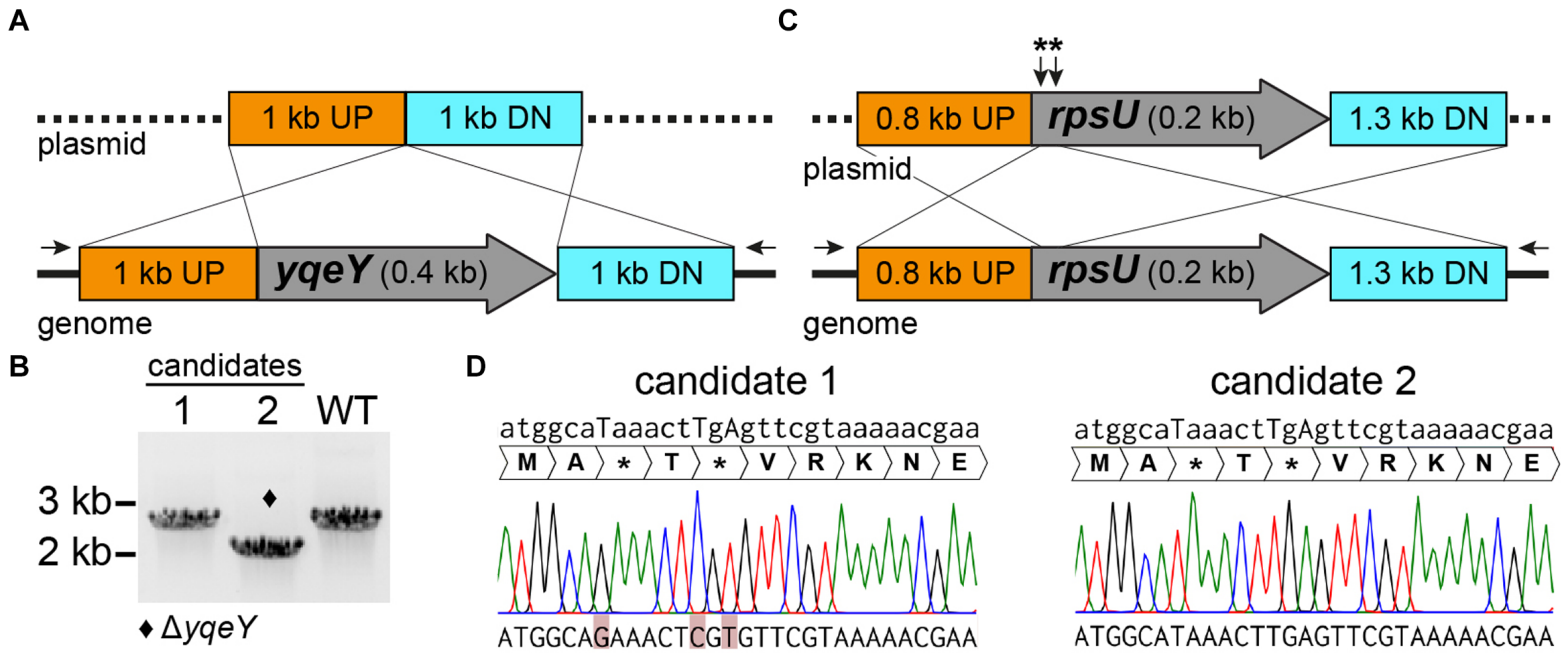
pBASE_Bha allelic replacement generates chromosomal deletions and point mutations in *H. halodurans*. **(A)** Construct design for the pBASE_Bha_ΔyqeY plasmid payload region containing 1 kb flanking regions upstream (UP) and downstream (DN) of *yqeY*, to delete *yqeY* from the genome via double crossover. Black arrows depict the location of the binding sites for PCR primers used to amplify the *yqeY* region exclusively from the genome to validate the desired deletion. **(B)** PCR screening for *yqeY* deletion mutants from two candidate colonies. The respective *yqeY* region was amplified with primers shown in (A), wherein the desired deletion yields a shorter product (2 kb) compared to the full-length PCR amplification product (2.4 kb) from wild-type (WT) bacteria. Successful deletion of *yqeY* is indicated by the diamond symbol. **(C)** Construct design for the pBASE_Bha_rpsU** plasmid payload region containing the flanking regions upstream (UP) and downstream (DN) of *rpsU*, to introduce three point mutations to yield two early stop codons (**). **(D)** Sequencing chromatograms of two *rpsU* mutation candidates. Position of point mutations are indicated by uppercase letters in the desired mutant sequence above chromatograms. Unsuccessful introduction of mutations (i.e., *rpsU* wild-type sequence) in the actual sequenced DNA at the bottom of the figure is highlighted in red for candidate 1, whereas candidate 2 carries the desired mutations.

Notably, the number of candidates per screening (clones that only grow on plain agar) as well as the ratio of successfully chromosomally modified clones to clones with the wild-type sequence are highly variable and seem to depend on the target sequence. Of candidates which underwent the second homologous recombination event and have eliminated the plasmid from the chromosome, 4–100% had the desired knockout, depending on the gene to be deleted. The remaining clones were wild type for the targeted gene (Table 3). We note that the theoretical likelihood either to obtain the desired mutation or to restore the wild-type allele via the second homologous recombination event is 50% each, due to the nature of the procedure. Therefore, ensuing screening via PCR is required (step 7, Figure 3C). We present two examples using the above-described methods to illustrate the successful deletion of *H. halodurans yqeY* (BH1355, AYT26_RS07050) and insertion of point mutations into *rpsU* (BH1354, AYT26_RS07045) to introduce two early stop codons to functionally disrupt RpsU (ribosomal protein S21) (Figure 4). Successful deletion of the *yqeY* gene was confirmed by colony PCR analysis (Figure 4B) whereas successful introduction of mutations into the *rpsU* gene was confirmed by ensuing sequencing of the PCR products (Figure 4D).

## 4 Discussion

The methods described herein have been successfully used with *H. halodurans* C-125 to delete more than 20 different genes and to generate more than six specific point mutations (data not shown). The scarless deletion of genes enables the generation of multiple-gene knockouts in the same genome without the risk of loss of genomic sequences via unwanted secondary excision or recombination events. Another advantage is that the deletion of genes without insertion of an antibiotic resistance cassette allows the introduction of plasmids for complementation studies or for other uses. In an organism such as *H. halodurans* that favors growth under alkaline conditions, only a few antibiotics are available for experiments involving genetic selection because most antibiotics are inactive at high pH. Given the limited availability of useful antibiotics in this species, a method that does not require antibiotic resistance markers for making routine genetic alterations is particularly useful. Moreover, the clean deletion of genes reduces the chances for polar effects created by the insertion of an antibiotic resistance cassette (Mateus et al., 2021). If polar effects are still suspected due to the deletion of a gene, the introduction of early stop codons is now a suitable solution to obtain a functional knockout without drastically changing the genomic sequence or altering the length of the altered mRNA transcript.

In rare instances, *in vitro* methylation was observed to yield higher colony numbers per μg plasmid DNA than that observed from *in vivo* methylation (data not shown). Possibly, in these cases, the pPAMC125 (methylation vector) to target vector ratio is not 1:1. If true, transformation of 2 μg plasmid DNA would deliver less than the expected 1 μg of target vector DNA. In contrast to the *in vivo* methylated plasmid preparation, which contains both plasmids, the *in vitro* methylation process yields only the target vector.

Notably, the type II DNA 5-cytosine methyltransferase encoded by BH3508 in the strain *H. halodurans* C-125 is part of a prophage and does not occur in other *H. halodurans* strains. Therefore, *in vitro* methylation with the HaeIII methyltransferase of *H. aegyptius* will likely only improve transformation efficiency in *H. halodurans* C-125. Nonetheless, the concept of *in vitro* plasmid methylation can be applied to other *H. halodurans* strains and other bacterial species if the respective methyltransferases are known and an enzyme with the same methylation-specificity is available. A recently developed method called IMPRINT (Imitating Methylation Patterns Rapidly IN TXTL) has been reported (Vento et al., 2024) that uses cell-free transcription-translation (TXTL) systems to *in vitro* methylate DNA in a manner that matches the expected methylation pattern of the species targeted for transformation. This approach overcomes the need to purchase or otherwise prepare the necessary methyltransferases.

In conclusion, the methods described herein permit the rapid and routine genetic manipulation of *H. halodurans* C-125, which enhances the use of this species as a model organism for the study of extremophilic bacteria. The pBASE_Bha vector should also be suitable for genetically modifying the *H. halodurans* isolate LB-1 (NCBI reference genome), as this strain only has a single mismatch in the *secY* antisense sequence (one out of 542 nucleotides) compared to the antisense sequence in the current vector. For more distantly related *H. halodurans* strains, it may be necessary to adapt the *secY* antisense sequence for use in the newly targeted genome. *S. aureus* and *H. halodurans* both belong to the order of *Bacillales*, but are members of different families (i.e., *Staphylococcaceae* and *Bacillaceae*, respectively) (Schoch et al., 2020). Therefore, the method of allelic replacement with inducible counter-selection originally developed for *S. aureus* (Bae and Schneewind, 2006; Geiger et al., 2012) might be more broadly applicable to Gram-positive species of the order of *Bacillales* because it was readily adapted to *H. halodurans* with only few modifications. Nevertheless, the method would need to be tested in more species of the order of *Bacillales* to demonstrate broader applicability.

## Data Availability

The original contributions presented in the study are included in the article/Supplementary material, further inquiries can be directed to the corresponding author.
