# Supplementary material for: Improved methods for genetic manipulation of the alkaliphile Halalkalibacterium halodurans
Source: Front Microbiol. 2024 Sep 18;15:1465811. doi: 10.3389/fmicb.2024.1465811 (PMC11445130; doi:10.3389/fmicb.2024.1465811)
Supplement: Supplementary file 1 [file Data_Sheet_1.PDF]

Plasmid sequence of pBASE_Bha (pFW004)

The DNA sequence of the 6,579 bp-long plasmid pBASE_Bha (pFW004) is detailed below. Genes, regulatory elements etc. are highlighted as described in the legend.

Legend:

Bold italic: Multiple Cloning Site (MCS)

Bold underlined: sequence of pBASE_MCS primers

Bold underlined pink and black: temperature sensitive origin of replication (*ori* pET194ts) which overlaps with the *cop6* element

Pink: *cop6* element

Blue*: repF* element

Cyan*: tet* repressor (*tetR*)

Bold: *tet* operator sequence (*tetO*)

Underlined: $P_{xyl/tet}$ promoter (contains a *tetO* sequence)

Green lower-case letters: *H. halodurans secY* antisense sequence (Bha *secY* antisense)

Orange underlined: chloramphenicol resistance cassette (chloramphenicol acetyltransferase, *cat*)

Bold purple: Gram-negative origin of replication (*ori ColE1*)

Orange: ampicillin resistance cassette (β-lactamase, *bla*)

>>>/<<< indicates gene or element orientation

Plasmid DNA sequence:

Multiple Cloning Site (MCS)
GAATTCCGGAGCTCGGTACCCGGGCTAGCGCGCAGATCTGTCGACGATATCAAGCTTGCATGCCTGC

AGAACGGATTGTTGATGATTACGAAAATATTAAGAGCACAGACTATTACACAGAAAATCAAGAATTAAAAAAA

<<<pBASE_MCS_R (primer)
CGTA**GAGAGAGTTTGAAAGAAGTAGTGAATACATGG**AAAGAGGGGTATCACGAAAAAAGTAAAGAGGTTA

ATAAATTAAAGCGAGAGAATGATAGTTTGAATGAGCAGTTGAATGTATCAGAGAAATTTCAAGATAGTACAGT

GACTTTATATCGTGCTGCGAGGGCGAATTTCCCTGGGTTTGAGAAAGGGTTTAATAGGCTTAAAGAGAAATT

CTTTAATGATTCCAAATTCGAGCGTGTGGGACAGTTTATGGATGTTGTACAGGATAATGTCCAGAAGGTCGAT

AGAAAGCGTGAGAAACAGCGTACAGACGATTTAGAGATGTAGAGGTACTTTTATGCCGAGAAAACTTTTTG

CGTGTGACAGTCCTTAAAATATACTTAGAGCGTAAGCGAAAGTAGTAGCGACAGCTATTAACTTTCGGTTGC

AAAGCTCTAGGATTTTTAATGGACGCAGCGCATCACACGCAAAAAGGAAATTGGAATAAATGCGAAATTTG

ori pET194ts>>>
AGATGTTAATTAAAGACCTTTTTGAGGTCTTTTTTTCTTAGATTTTTGGGGTTATTTA**GGGGAGAAAACATAGG**

GGGGTACTACGACCTCCCCCCTAGGTGTCCATTGTCCATTGTCCAAACAAATAAATAAATATTGGGTTT

cop6>>>
TTAATGTTAAAAGGTTGTTTTTTATGTTAAAGTGAAAAAAACAGATGTTGGGAGGTACAGTGATgGTTGTAG

ATAGAAAAGAAGAGAAAAAAGTTGCTGTTACTTTAAGACTTACAACAGAAGAAAATGAGATATTAAATAG

AATCAAAGAAAAATATAATATTAGCAAATCAGATGCAACCGGTATTCTAATAAAAAAATATGCAAAGGAGGA

ATACGGTGCATTTTAAACAAAAAAAGATAGACAGCACTGGCATGCTGCCTATCTATGACTAAATTTTGTTAAG

repF>>>
TGTATTAGCACCGTTATTATATCATGAGCGAAAATGTAATAAAAGAAACTGAAAACAAGAAAAATTCAAGAGG

ACGTAATTGGACATTTGTTTTATATCCAGAATCAGCAAAAGCCGAGTGGTTAGAGTATTTAAAAGAGTTACAC

ATTCAATTTGTAGTGTCTCCATTACATGATAGGGATACTGATACAGAAGGTAGGATGAAAAAAGAGCATTATC

ATATTCTAGTGATGTATGAGGGTAATAAATCTTATGAACAGATAAAAATAATTACAGAAGAATTGAATGCGACTA

TTCCGCAGATTGCAGGAAGTGTGAAAGGTCTTGTGAGATATATGCTTCACATGGACGATCCTAATAAATTTAA

ATATCAAAAAGAAGATATGATAGTTTATGGCGGTGTAGATGTTGATGAATTATTAAAGAAAACAACAACAGATA

GATATAAATTAATTAAAGAAATGATTGAGTTTATTGATGAACAAGGAATCGTAGAATTTAAGAGTTTAATGGATTA

TGCAATGAAGTTTAAATTTGATGATTGGTTCCCGCTTTTATGTGATAACTCGGCGTATGTTATTCAAGAATATAT

AAAATCAAATCGGTATAAATCTGACCGATAGATTTTGAATTTAAGAGTGTCACAAGACACTCTTTTTTCGCAC

CAGCGAAAACTGGTTTAAGCCGACTGCGCAAAAGACATAATCGATTCACAAAAAATAGGCACACGAAAAA

CAAGTTAAGGGATGCAGTTTATGCATCCCTTAACTTACTTATTAAATAATTTATAGCTATTGAAAAGAGATAAGA

ATTGTTCAAAGCTAATATTGTTTAAATCGTCAATTCCTGCATGTTTTAAGGAATTGTTAAATTGATTTTTTGTAAAT

ATTTTCTTGTATTCTTTGTTATCTTGGTTACCGTGAAGTTACCATCACGGAAAAAGGTTATGCTGCTTTTAAGAC

CCACTTTCACATTTAAGTTGTTTTTCTAATCCGCATATGATCAATTCAAGGCCGAATAAGAAGGCTGGCTCTG

CACCTTGGTGATCAAATAATTCGATAGCTTGTCGTAATAATGGCGGCATACTATCAGTAGTAGGTGTTTCCCT

TTCTTCTTTAGCGACTTGATGCTCTTGATCTTCCAATACGCAACCTAAAGTAAAATGCCCCACAGCGCTGAG

TGCATATAATGCATTCTCTAGTGAAAAACCTTGTTGGCATAAAAAGGCTAATTGATTTTCGAGAGTTTCATACT

GTTTTTCTGTAGGCCGTGTACCTAAATGTACTTTTGCTCCATCGCGATGACTTAGTAAAGCACATCTAAAACT

TTTAGCGTTATTACGTAAAAAATCTTGCCAGCTTTCCCCTTCTAAAGGGCAAAAGTGAGTATGGTGCCTATCT

AACATCTCAATGGCTAAGGCGTCGAGCAAAGCCCGCTTATTTTTTACATGCCAATACAATGTAGGCTGCTC

TACACCTAGCTTCTGGGCGAGTTTACGGGTTGTTAAACCTTCGATTCCGACCTCATTAAGCAGCTCTAATG

<<<tetR ***tetO>>>***

CGCTGTTAATCACTTTACTTTTATCTAATCTAGACATCATTAATTCCTCCTTTTTGTTGAC**ACTCTATCATTGATA**

$P_{xyl/tet}$>>>

GAGTTATTTGTCAAACTAGTTTTTTATTTGGATCCCCTCGAGTTCATGAAAAACTAAAAAAAATATTGAC**ACTC**

Bha *secY* antisense>>>

TATCATTGATAGAGTATAATTAAAATAAGCTTGATGGGtttccgactcccttagctgtaatctgctccccgagccacattaag

aaagccgttcctgcagtaaggacaagtgcgataaagagatacaccgacacgcttggattaggaattaatcctgggaagaagttgttaaaa

ccaaccgacatcccgagtgcctgaataaaccctaaaacaatcgttccatagcgggtaaattgagctagcttacgacgccctgcttcgcc

ctctttcgcccactcagcaaatttcggaacgacatccatctgcaataattgcatgacaatggatgctgtaatgtatggcatgatccccatcg

caaagatggagaaattcccaagtgctcctccgccaaacgtatttaaaaaaccgaatgcatttgcctgatcgacaaaatctagcacttctc

ggtttgtacctggaacggggataaaacttccgatccgaaaaacgatgagcatgagcagggtgaaaatgaccttacggcgcaaatcaccc

actcgaaaaatgttggagatcgttcggaacattagatcacctcagttCCTTAAGGGTAACTAGCCTCGCCGGCAATAGTTA

CCCTTATTATCAAGATAAGAAAGAAAAGGATTTTTCGCTACGCTCAAATCCTTTAAAAAAACACAAAAGACC

ACATTTTTTAATGTGGTCTTTATTCTTCAACTAAAGCACCCATTAGTTCAACAAACGAAAATTGGATAAAGTGG

GATATTTTTAAAATATATATTTATGTTACAGTAATATTGACTTTTAAAAAAGGATTGATTCTAATGAAGAAAGCAGA

cat>>>

CAAGTAAGCCTCCTAAATTCACTTTAGATAAAAATTTAGGAGGCATATCAAATGAACTTTAATAAAATTGATTTA

GACAATTGGAAGAGAAAAGAGATATTTAATCATTATTTGAACCAACAAACGACTTTTAGTATAACCACAGAAA

TTGATATTAGTGTTTTATACCGAAACATAAAACAAGAAGGATATAAATTTTACCCTGCATTTATTTTCTTAGTGAC

AAGGGTGATAAACTCAAATACAGCTTTTAGAACTGGTTACAATAGCGACGGAGAGTTAGGTTATTGGGATAA

GTTAGAGCCACTTTATACAATTTTTGATGGTGTATCTAAAACATTCTCTGGTATTTGGACTCCTGTAAAGAATGA

CTTCAAAGAGTTTTATGATTTATACCTTTCTGATGTAGAGAAATATAATGGTTCGGGGAAATTGTTTCCCAAAA

CACCTATACCTGAAAATGCTTTTTCTCTTTCTATTATTCCATGGACTTCATTTACTGGGTTTAACTTAAATATCAA

TAATAATAGTAATTACCTTCTACCCATTATTACAGCAGGAAAATTCATTAATAAAGGTAATTCAATATATTTACCG

CTATCTTTACAGGTACATCATTCTGTTTGTGATGGTTATCATGCAGGATTGTTTATGAACTCTATTCAGGAATTG

TCAGATAGGCCTAATGACTGGCTTTTATAATATGAGATAATGCCGACTGTACTTTTTACAGTCGGTTTTCTAAT

GTCACTAACCTGCCCCGTTAGTTGAAGAAGGTTTTTATATTACAGCTCCAGATCCATATCCTTCTTTTTCTGAA

CCGACTTCTCCTTTTTCGCTTCTTTATTCCAATTGCTTTATTGACGTTGAGCCTCGGAACCGGCATGTGAGC

AAAAGGCCAGCAAAAGGCCAGGAACCGTAAAAA**GGCCGCGTTGCTGGCGTTTTTCCATAGGCTCCGC**

CCCCCTGACGAGCATCACAAAAATCGACGCTCAAGTCAGAGGTGGCGAAACCCGACAGGACTATAA

AGATACCAGGCGTTTCCCCCTGGAAGCTCCCTCGTGCGCTCTCCTGTTCCGACCCTGCCGCTTACC

GGATACCTGTCCGCCTTTCTCCCTTCGGGAAGCGTGGCGCTTTCTCATAGCTCACGCTGTAGGTATCT

CAGTTCGGTGTAGGTCGTTCGCTCCAAGCTGGGCTGTGTGCACGAACCCCCCGTTCAGtCCGACCG

CTGCGCCTTATCCGGTAACTATCGTCTTGAGTCCAACCCGGTAAGACACGACTTATCGCCACTGGCA

GCAGCCACTGGTAACAGGATTAGCAGAGCGAGGTATGTAGGCGGTGCTACAGAGTTCTTGAAGTGGT

GGCCTAACTACGGCTACACTAGAAGGACAGTATTTGGTATCTGCGCTCTGCTGAAGCCAGTTACCTTC

GGAAAAAGAGTTGGTAGCTCTTGATCCGGCAAACAAACCACCGCTGGTAGCGGTGGTTTTTTTGTTTG

CAAGCAGCAGATTACGCGCAGAAAAAAAGGATCTCAAGAAGATCCTTTGATCTTTTCTACGGGGTCTG

<<<ori ColE1
ACGCTCAGTGGAACGAAAACTCACGTTAAGGGATTTTGGTCATGAGATTATCAAAAAGGATCTTCACCTA

GATCCTTTTAAATTAAAAATGAAGTTTTAAATCAATCTAAAGTATATATGAGTAAACTTGGTCTGACAGTTACCA

ATGCTTAATCAGTGAGGCACCTATCTCAGCGATCTGTCTATTTCGTTCATCCATAGTTGCCTGACTCCCCGT

CGTGTAGATAACTACGATACGGGAGGGCTTACCATCTGGCCCCAGTGCTGCAATGATACCGCGAGACCC

ACGCTCACCGGCTCCAGATTTATCAGCAATAAACCAGCCAGCCGGAAGGGCCGAGCGCAGAAGTGGT

CCTGCAACTTTATCCGCCTCCATCCAGTCTATTAATTGTTGCCGGGAAGCTAGAGTAAGTAGTTCGCCAGTT

AATAGTTTGCGCAACGTTGTTGCCATTGCCGCAGGCATCGTGGTGTCACGCTCGTCGTTTGGTATGGCTTC

ATTCAGCTCCGGTTCCCAACGATCAAGGCGAGTTACATGATCCCCCATGTTGTGCAAAAAAGCGGTTAGC

TCCTTCGGTCCTCCGATCGTTGTCAGAAGTAAGTTGGCCGCAGTGTTATCACTCATGGTTATGGCAGCACT

GCATAATTCTCTTACTGTCATGCCATCCGTAAGATGCTTTTCTGTGACTGGTGAGTACTCAACCAAGTCATTC

TGAGAATAGTGTATGCGGCGACCGAGTTGCTCTTGCCCGGCGTCAATACGGGATAATACCGCGCCACAT

AGCAGAACTTTAAAAGTGCTCATCATTGGAAAACGTTCTTCGGGGCGAAAACTCTCAAGGATCTTACCGCT

GTTGAGATCCAGTTCGATGTAACCCACTCGTGCACCCAACTGATCTTCAGCATCTTTTACTTTCACCAGCGT

TTCTGGGTGAGCAAAAACAGGAAGGCAAAATGCCGCAAAAAAGGGAATAAGGGCGACACGGAAATGTT
<<<bla
GAATACTCATACTCTTCCTTTTTCAATATTATTGAAGCATTTATCAGGGTTATTGTCTCATGAGCGGATACATATT

TGAATGTATTTAGAAAAATAAACAAATAGGGGTTCCGCGCACATTTCCCCGAAAAGTGCCACCTGACGTCT

AAGAAACCATTATTATCATGACATTACCTATAAAAATAGGCGTATCACGAGGCCCTTTCGTCTTCAAGAATTT

ATTCTAGCTAGAGCGGCGGATTTGTCCTACTCAGGAGAGCGTTCACCGACAAACAACAGATAAAACGAAA
pBASE_MCS_F (primer)>>>
GGCCCAGTCTTTCGACTGAGCCTTTCGTTTTATTT**GATGCCTCAAGCTAGAGAGTCATTACC**CCAGGCGT

TTAAGGGCACCAATAACTGCCTTAAAAAAATTACGCCCCGCCCTGCCACTCATCGCAGTGCAGCG
